# Cooperation enhanced by the coevolution of teaching activity in evolutionary prisoner's dilemma games with voluntary participation

**DOI:** 10.1371/journal.pone.0193151

**Published:** 2018-02-16

**Authors:** Chen Shen, Chen Chu, Yini Geng, Jiahua Jin, Fei Chen, Lei Shi

**Affiliations:** 1 School of Statistics and Mathematics, Yunnan University of Finance and Economics, Kunming, Yunnan, PR China; 2 Library, Yunnan Normal University, Kunming, Yunnan, PR China; Southwest University, CHINA

## Abstract

Voluntary participation, as an additional strategy involved in repeated games, has been proved to be an efficient way to promote the evolution of cooperation theoretically and empirically. Besides, current studies show that the coevolution of teaching activity can promote cooperation. Thus, inspired by aforementioned above, we investigate the effect of coevolution of teaching activity on the evolution of cooperation for prisoner’s dilemma game with voluntary participation: when the focal player successfully enforces its strategy on the opponent, his teaching ability will get an increase. Through numerical simulation, we have shown that voluntary participation could effectively promote the fraction of cooperation, which is also affected by the value of increment. Furthermore, we investigate the influence of the increment value on the density of different strategies and find that there exists an optimal increment value that plays an utmost role on the evolutionary dynamics. With regard to this observation, we unveil that an optimal value of increment can lead to strongest heterogeneity in agents’ teaching ability, further promoting the evolution of cooperation.

## Introduction

How to understand the emergence and maintenance of cooperation in the context of natural selection remains a formidable challenge met by scientists from many different fields of natural and social sciences [1]. Evolutionary game theory provides a common mathematical framework to investigate this puzzle within groups of selfish individuals. In particular, the prisoner’s dilemma game (PDG), as a metaphor for capturing social dilemma, has drawn continuous attention to explore the possibilities enhancing the cooperative behavior among selfish individuals [2–5].

In the original PDG, each player decides whether to cooperate (C) or defect (D) simultaneously. They both receive reward *R* for mutual cooperation and punishment *P* for mutual defection. If a defector encounters a cooperator, the former will get a temptation to defect *T*, while the latter receives a sucker’s payoff *S*. These parameters satisfy: *T*>*R*>*P*>*S* and 2*R*>*T*+*S*. Obviously, defection is the best choice regardless of the opponent’s choice and hence leads to the tragedy of the commons, where private interest is at odds with the collective welfare [6].

In order to resolve this dilemma, a variety of scenarios have been proposed to offset the above unfavorable outcome and enhance the evolution of cooperation [7–15]. Nowak attributed all these works to five mechanisms: kin selection, group selection, direct reciprocity, indirect reciprocity and network reciprocity [16]. Particularly, network reciprocity, where players are constrained to play only with their direct neighbors, has been pioneered by Nowak and May and inspired a great deal of attention [17], for example, different network topologies: small-world network [18], random regular graph [19], BA scale-free network [20] multilayer network [21, 22], interdependent network [23] and so on. In addition, different factors have also been considered for exploring their impact on the evolution of cooperation. For instance, punishment [24–26], reward [27–29], reputation [30, 31], conformist [32, 33], to name but a few, all allow the emergence and persistence of cooperation even if the temptation to defect is large. What’s more, the impact of the teaching activity on the evolution of cooperation has been extensively explored and reached fruitful achievements [34–38]. For example, Szolnoki and Szabo (2007) study the quenched inhomogeneous distribution of teaching activity in evolutionary dynamics [34]; Szolnoki and Perc (2008) investigate the evolutionary games where the teaching activity of players can evolve in time and find an optimal value of the increment, which can best support the maintenance of cooperation [35]; Further they separately consider coevolution affecting either only the cooperators or only the defectors, and show that both options promote cooperation. Interestingly, they reveal that the co-evolutionary promotion of players spreading defection is more beneficial for cooperation [36].

Voluntary participation, as a third strategy except for cooperation and defection, has been turned out to be a simple yet effective way to promote the persistence of cooperative behavior [39, 40]. Specifically, when a player repugnance to risk would like to obtain a small but fixed payoff, he can refuse to participate in the PDG and choose the strategy as loner (*L*). The introduction of loner strategy would rather not lead the system falling into the homogeneous defection state but a rock-scissors-paper dynamics with cyclic dominance. From aforementioned above, we can impose an interesting question: how cooperation fares when the teaching activity is considered into prisoner’s dilemma games with voluntary participation? The rest of this paper are organized as follows: we first describe our modified model of PDG with voluntary participation; subsequently, the main simulation results are shown in Section 3; lastly, we summarize our conclusions in Section 4.

## Results

**Fig 1:**
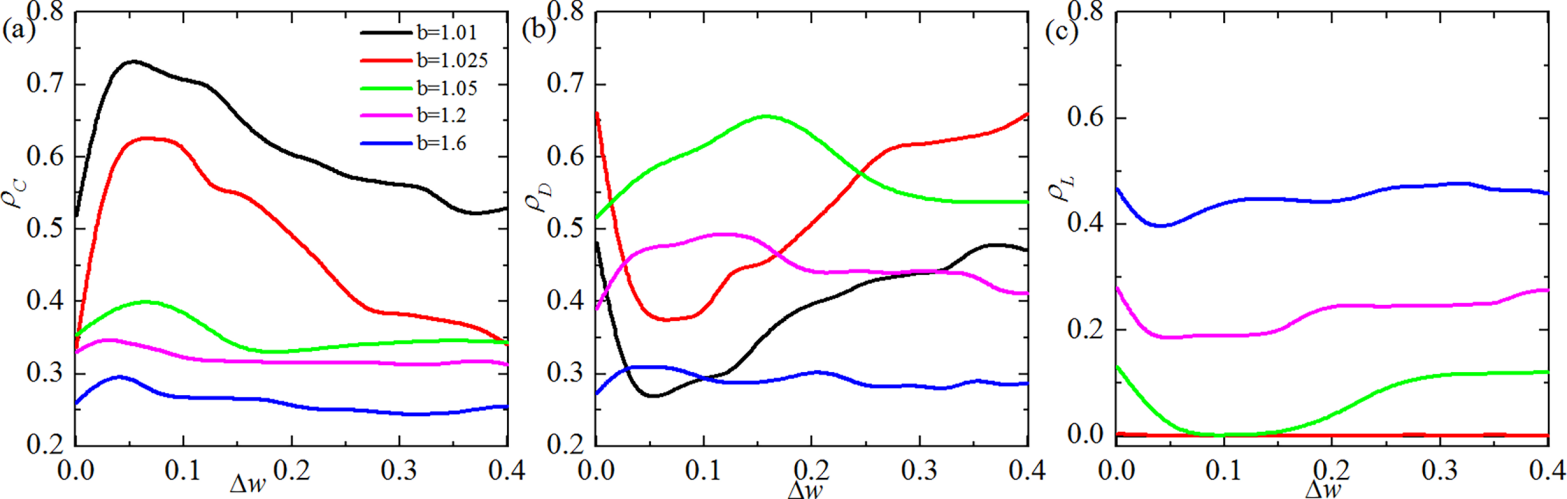
The frequencies of coopertors (Fig 1(A)), defectors (Fig 1(B)) and loners (Fig 1(C)) as a function of Δ for different values of temptation to defect *b*. All results are obtain for *K* = 0.1.

We first study the relationship between fraction of cooperation and Δ*w*, fraction of defection and Δ*w*, as well as fraction of loner and Δ*w* for different values of temptation to defect *b* in Fig 1(A), Fig 1(B) and Fig 1(C), respectively. With a fast scan of these results, we find that there exists a moderate Δ*w*, which can lead the fraction of cooperation reaching the maximum value no matter what value of *b* is applied. When the temptation to defect *b* is sufficiently small (i.e. *b* = 1.01 or *b* = 1.025), regardless of Δ*w*, only cooperators and defectors can exist in the equilibrium. Besides, when Δ*w* takes the intermediate value (Δ*w* ≈ 0.05), the fraction of cooperation can reach its maximum value, while the fraction of defection would have a minimum value. When *b* is relatively large, such as *b* = 1.05, *b* = 1.2 or *b* = 1.6, three strategies coexist in network. Interestingly, the fraction of cooperators, defectors could reach its maximum value, however, the fraction of loners has the minimum value around the moderate value of Δ*w*. For small *b*, the effect of enhanced network reciprocity can enable the cooperators to survive and even domain. With *b* continues to increase, the effect of enhanced network reciprocity would be weakened and cooperators need loners to protect them from being wiped out on the dynamic process. Thus three strategies fall into cycle dominance. Interestingly, the cooperation behaviors for Δ*w* = 0 and Δ*w* = 0.4 are nearly the same no matter what value of *b* applies. In fact, for the case of Δ*w* = 0, the teaching ability of players will not evolve, and thus the system is homogeneous. However, although the teaching ability of players will evolve for large Δ*w*, the system essentially leave the whole population in a homogeneous state for the too fast stop of the teaching ability, whereas a very few influential players having *w*_*x*_ close to 1.

**Fig 2:**
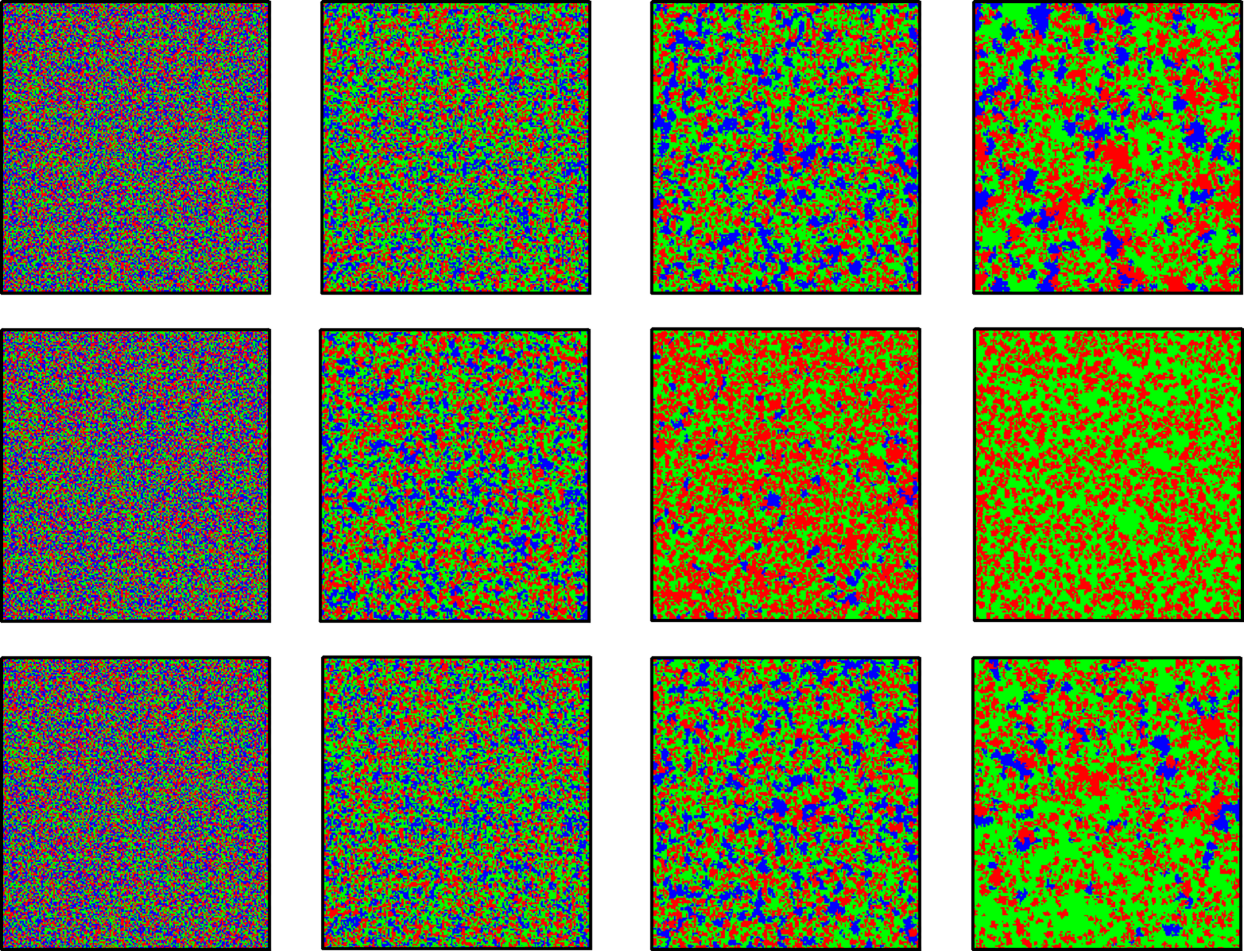
Typical snapshots of the distribution of strategies in step 0, 100, 800, 10,000. All results are obtain for *b* = 1.05 *K* = 0.1. From top to bottom, Δ*w* are equal to 0, 0.05 and 0.3 respectively. Cooperation, defection and loner are colored by red, green and blue, respectively.

In order to further validate the above results, some typical evolution snapshots of clusters have been shown in Fig 2. From top to bottom, Δ*w* are equal to 0, 0.05 and 0.3, and the time steps from left to right are equal to 0, 100, 800, 10000. Since cooperators dominate loners, loners dominate defectors and defectors dominate cooperators, three strategies coexist in the system at the stable state for traditional game with voluntary participation. However, from the second panel (Δ*w* = 0.05), we can see that loners go extinct and cooperators can survive by forming compact clusters to resist the invasion of defectors due to the enhanced network reciprocity. When Δ*w* is sufficiently large (lower panel), the system will fall into the cycle dominance of three strategies again, which is due to the weakening enhanced network reciprocity affected by too fast stop of the evolution of teaching ability.

**Fig 3:**
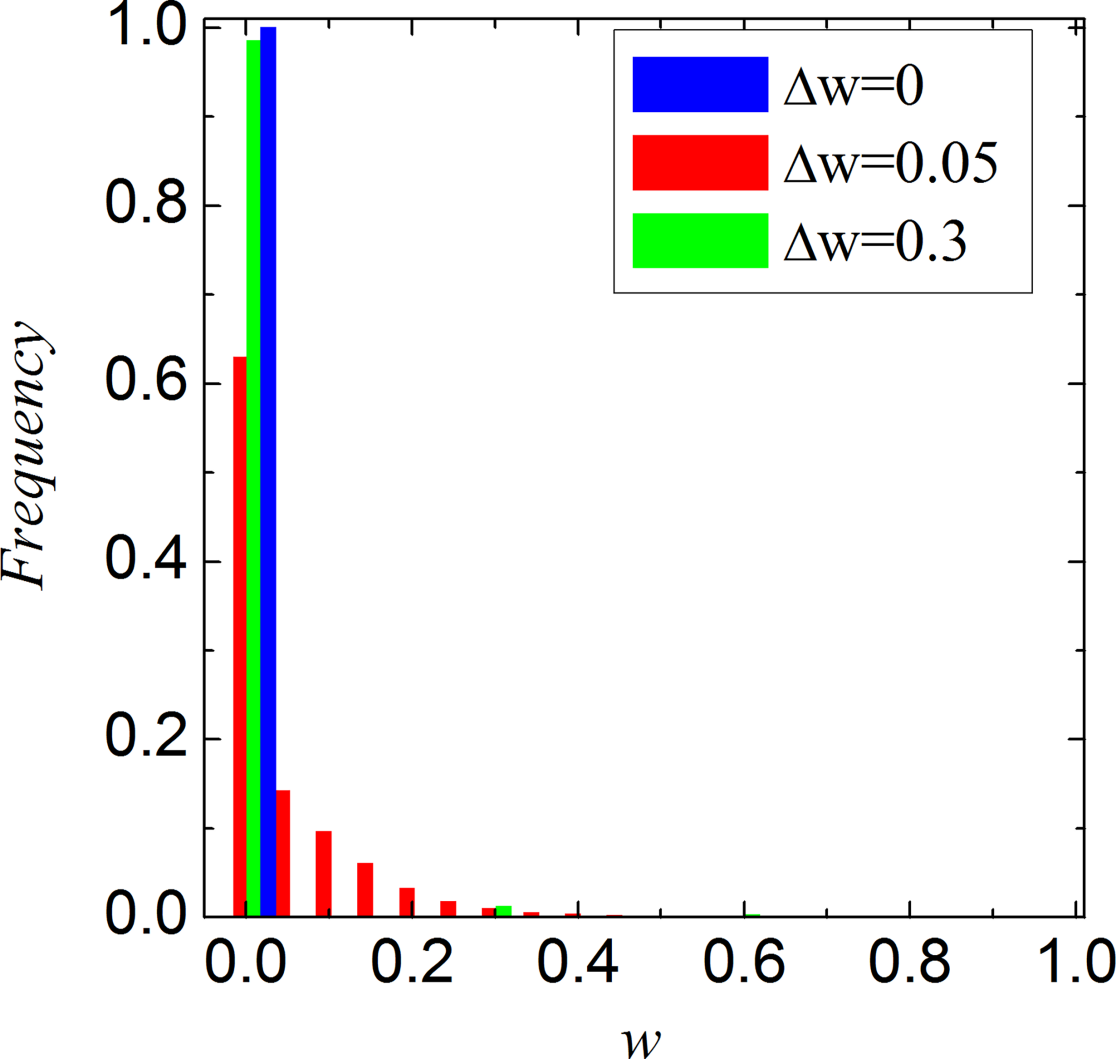
Stable distribution of teaching ability when *b* = 1.05 and *K* = 0.1.

From the observation of Fig 1 and Fig 2, we can see that a moderate value of Δ*w* can best promote cooperation. For the sake of exploring the potential reason of these results, Fig 3 features the distribution of player’s teaching ability in stable state. It is obvious that the teaching ability is a fixed value when Δ*w* = 0. However, when Δ*w* > 0, an interesting phenomenon appears: the teaching ability of player is no longer a fixed value, but exhibits heterogeneity which is introduced by our coevolution setup. Furthermore, the heterogeneity of players leads to an enhanced network reciprocity and further promotes the evolution of cooperation. In fact, the variance of teaching ability for different Δ*w* in Fig 3 are equal to 0, 0.08, 0.05, respectively. That is to say the heterogeneity of players’ teaching ability is largest when Δ*w* = 0.05. Previous works has proved that the positive effect of heterogeneity on the evolution of cooperation, such as heterogeneity network [41–43], social diversity [44, 45], heterogeneity aspirations [46], to name only a few examples. It is easy to understand why there exists a moderate value of Δ*w* that can best promote the evolution of cooperation in structured population.

**Fig 4:**
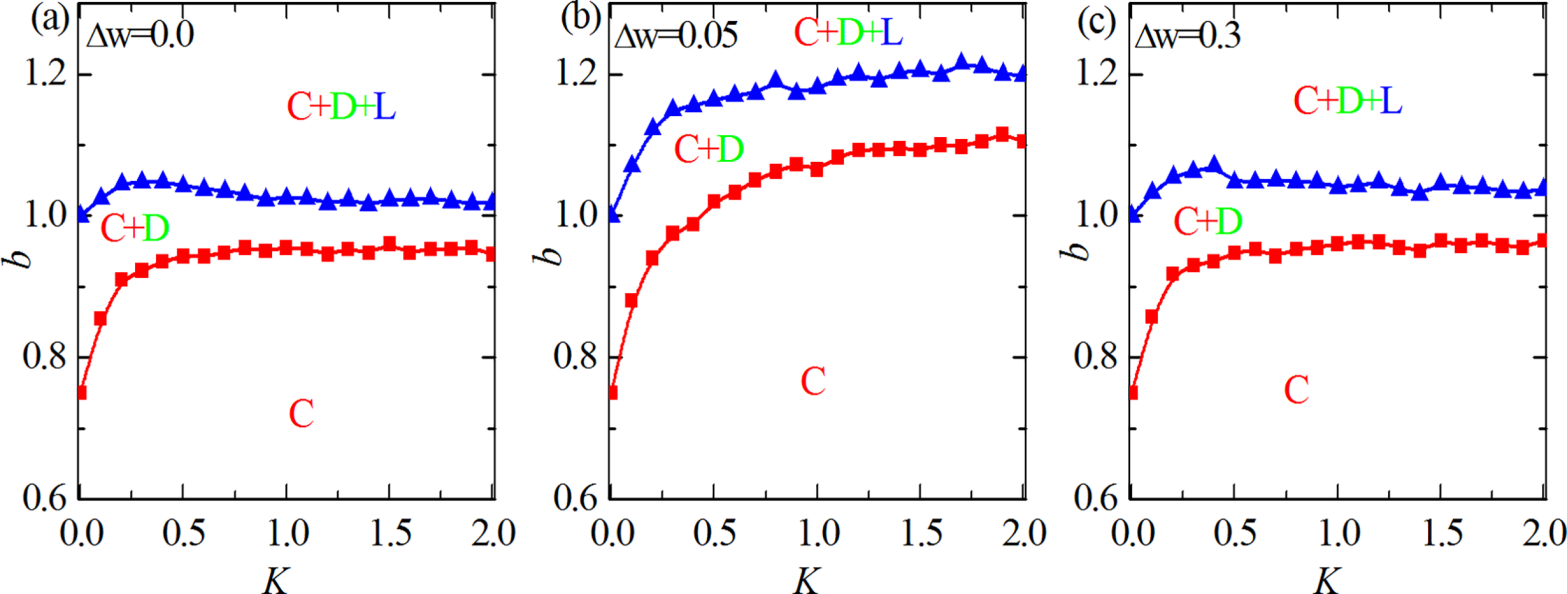
Phase separation lines on *K*-*b* parameter plane for different values of Δ*w*. From (a) to (c), the value of Δ*w* are equal to 0, 0.05 and 0.3, respectively. When Δ*w* is moderate, the space of pure C become wider and the space of mixed C+D+L become narrow, besides, the optimal uncertainty is disappear.

Finally, it is instructive to examine the evolution of cooperation under different levels of uncertain *K* by strategy adoptions. When *K* → ∞, all information is lost, players switch to neighbor’s strategy completely at random, while *K* → 0 enables the complete deterministic selections of the neighbor’s strategy. Fig 4 shows the phase transition lines on the full *K* − *b* parameter plane for different values of Δ*w*. Fig 4(A) (Δ*w* = 0) features a bell shaped phase boundary separating the pure *C* and mixed *C* +*D* + *L* phases, implying that an optimal level (*K* ≈ 0.25) of uncertainty can best promote cooperation. When Δ*w* is moderate (i.e. Δ*w* = 0.05), the space of pure *C* phases become wider and mixed *C* +*D* + *L* phases become narrow, besides, the optimal uncertainty disappears. Namely, the larger the value of *K*, the higher level of cooperation. However, when Δ*w* is sufficiently large (i.e. Δ*w* = 0.3), the space of pure *C* become narrow and mixed *C* +*D* + *L* become wider again, what’s more, the optimal uncertainty recovers. Nevertheless, from mentioned above, we can see that the consideration of coevolution of teaching ability and strategy not only supports the presence of cooperation, but also guarantees the best environment for cooperation to survive.

## Conclusion

To conclude, we have studied the coevolution of teaching ability and strategy for prisoner’s dilemma game with voluntary participation. Through numerical simulation, we have found that moderate value of Δ*w* can best promote the evolution of cooperation. Compared with the traditional game with voluntary participation, the heterogeneous distribution of player’s teaching ability is introduced into the system. Further, the enhanced network reciprocity occurs, which affects the evolution trend and promotes the evolution of cooperation. However, relatively large Δ*w* weakens the strength of the enhanced network reciprocity, leading the system falling into cycle dominance again. We hope our work can shed some meaningful lights on resolving the social dilemmas in realistic world.

## Models

In our work, each player can choose to be a cooperator (*s*_*x*_ = *C*), a defector (*s*_*x*_ = *D*), or a loner (*s*_*x*_ = *L*) in each round of the game. With regard to interaction network, we choose the square lattice with four direct neighbors of size *L* * *L*. Based on the weak PDG [39]: *R* = 1, *P* = *S* = 0, and *T* = *b*. The payoffs for two players in the prisoner’s dilemma game with voluntary participation can be represented as the matrix:

$$A=\left(\begin{array}{ccc} 1 & 0 & \delta \\ b & 0 & \delta \\ \delta & \delta & \delta \end{array}\right),$$ (1)

where *b* (1 < *b* ≤ 2) represents the temptation to defect and *δ* ∈ (0, 1) denotes the payoff of both the risk averse loner and its opponent. For simplicity but without loss of generality, *δ* is fixed to be 0.3 [47].

The game is iterated forward in accordance with Monte Carlo (MC) simulation composed of the following elementary steps. First, a randomly selected player *x* evaluates his benefits *p*_*x*_ by interacting with his direct neighbors. Next, player *x* choose one neighbor at random, say *y*, who also gets his payoff *p*_*y*_ in the same way. Finally, player *x* tries to enforce its strategy *s*_*x*_ on player *y* in accordance with the modified fermi function:

$$W=w_{x}\;*\frac{1}{1+\exp\left[(p_{y}-p_{x}\right)/K]},$$ (2)

Where *K* denotes the amplitude of noise or its inverse the so-called intensity of selection [40]. Besides, *w*_*x*_ represents the ability of the transfer strategy of player *x* (teaching activity of player *x*). Following Szolnoki and Perc [35], the teaching activity *w*_*x*_ changes adaptively in time as follows. Initially, all players are given the minimal value *w*_*x*_ = 0.01 throughout this paper in order to avoid frozen states. Next, the pre factor *w*_*x*_ will be increased by a constant positive value Δ*w* when the focal player *x* succeeds in enforcing its strategy on his opponent *y* for each time step. Lastly, the evolution of *w*_*x*_ is stopped as soon as one *w*_*x*_ reaches 1. It is worth mentioning that our work is different from the previous work [35], we mainly apply the setup of coevolution of strategy and teaching ability in that work to our PDG with voluntary participation, and investigate the evolution of cooperation.

MC results presented below are obtained on population comprising 400*400 individuals and the stationary fractions of these strategies are determined with 5 * 10^3^ steps after sufficiently long transients. Moreover, since the teaching activity may introduce additional disturbances, the final results have been averaged over up to 10 independent realizations for each set of parameter values in order to assure suitable accuracy.

## Supporting information

S1 File This file (zip format) contains the raw data used in figures with MC simulation. (RAR)
